# Perceived socially responsible-HRM on talent retention: The mediating effect of trust and motivation and the moderating effect of other-regarding value orientation

**DOI:** 10.3389/fpsyg.2022.1087065

**Published:** 2023-01-16

**Authors:** Zainab Ali Rawshdeh, Zafir Khan Mohamed Makhbul, Mustafa Rawshdeh, Suguna Sinniah

**Affiliations:** ^1^Department of Management, College of Business Administration, Prince Sultan University, Riyadh, Saudi Arabia; ^2^Graduate School of Business, Universiti Kebangsaan Malaysia, Bangi, Malaysia; ^3^Department of Industrial Engineering, Faculty of Engineering, The Hashemite University, Zarqa, Jordan

**Keywords:** perceived socially responsible-HRM, talent retention, trust, motivation, other-regarding value orientation, structural equation mode (SEM)

## Abstract

**Introduction:**

Studies have shown that there is a growing interest in corporate social responsibility (CSR) and talent management, and the identified links between their aspects. Thus, the current study examined the relationship between socially responsible human resource management (SRHRM)–identified as CSR strategies and practices directed at employees to underpin the effectiveness of CSR implementation–and talent retention. In addition, this study employed a mediation-moderation framework with employee attitude (motivation and trust) as a mediating variable and other-regarding value orientation (ORVO) as a moderating variable. Thus, the current study contributes to talent management and CSR current knowledge by analyzing the depth of the relationship by way of exploring the moderating and mediating process. It answers the “how” and “when” questions and explains the mechanism through which an organization can use its socially responsible HRM practices for retaining talented employees.

**Methods:**

A total of 418 people from the Klang Valley area in Malaysia participated in the study. The hypotheses in the study were tested using PLS structural equation modeling.

**Results:**

The results of the study showed that (a) perceived SRHRM was positively related to talent retention, (b) this relationship was partially mediated by the employee’s attitude (motivation and trust), and (c) ORVO did not moderate the relationship between perceived SRHRM and talent retention.

**Discussion:**

Furthermore, the study findings provide concrete and actionable recommendations on how to formulate and implement appropriate SRHRM policies and practices as they are not only essential for the successful implementation of external CSR programs but also essential for retaining talented employees and for improving their motivation and organizational trust.

## 1. Introduction

According to HR directors in Europe, talent management has become the most significant challenge for CEOs, with only 30% of them seeing that they have the talent they require to attain their future development ambitions (Stahl et al., 2012). Meanwhile, in the USA, talent is among the top three priorities for US CEOs (Groysberg and Connolly, 2015). Dobbs et al. (2012) reported that there will be a scarcity of talented workforce, and even the demand for them will be pivotal in the coming years (Michaels et al., 2014). Accordingly, the ability to attract and retain talent is rapidly becoming one of the core competencies of high-performance organizations in both developed and emerging countries, in addition to the fundamental concern for organization leaders (Bartlett and Ghoshal, 2002; Glen, 2007). However, attracting talent is not enough; there must be a planned strategy for managing talents, supported by processes to retain their commitment and properly use their abilities (McDonnell, 2011; Khandelwal and Shekhawat, 2018). Hence, the interest in talent retention has been a serious concern for managers over the past decade (Beechler and Woodward, 2009; Collings and Mellahi, 2009; Skuza et al., 2013).

Corporate social responsibility (CSR) may be important in helping firms to explore how to improve talent retention (Farndale et al., 2010). A contemporary school of thought promotes the idea that there is solid proof demonstrating the growing importance of an organization’s CSR practices as a successful weapon and a significant factor in talent attraction and retention (Macey and Schneider, 2008; Farndale et al., 2010; Tymon et al., 2010; Camilleri, 2016). The links between aspects of talent management and CSR have been identified in several studies (Fombrun and Shanley, 1990; Greening and Turban, 2000; Tymon et al., 2010; Green et al., 2014). Furthermore, this growing interest in CSR is motivated by the talent’s preferences for organizations that reflect their values, pride for affiliation, and that provide a positive expectation regarding working conditions within the organization (Jones et al., 2014). CSR can create an effective tactic that positively impacts the processes of attracting talents, as well as motivating and retaining them (Pinkess, 2008; Camilleri, 2016), and helps the organization to improve talent retention (Farndale et al., 2010). This importance for CSR arises from society as an institutional regulation, as well as from business organizations to improve their public image and increase their profitability (Bertels and Peloza, 2008; Quazi et al., 2015).

Human resource management (HRM) plays a very critical role in the implementation of CSR and sustainable strategy in organizations (Bučiūnienė and Kazlauskaitė, 2012; Manroop et al., 2014). A growing number of organizations have started to employ HRM as a key factor to improve their socially responsible business performance and to prepare their managers for social, ecological, and ethical issues (Newman et al., 2016; Cohen, 2017). Orlitzky et al. (2006) introduced the term socially responsible human resource management (SRHRM). It is defined as strategies and practices of CSR toward employees to improve the effectiveness of CSR practices implementation (Shen and Benson, 2016). Furthermore, it may include taking into account the social contributions of the employees in the processes of recruitment, performance appraisal, promotion, remuneration, and retention, in addition to giving employees training programs on social issues (Orlitzky et al., 2006; Shen and Benson, 2016). SRHRM has a positive effect on employees’ attitudes and behaviors in the organization, such as their organization citizenship behavior, intention to quit, and employees’ work-related attitudes (trust, motivation, and affective commitment) (Kundu and Gahlawat, 2016a; Newman et al., 2016). Accordingly, scholars extended and combined CSR to the HRM foundation which promotes the current knowledge of both bodies of the literature. Therefore, using a measure of socially responsible HRM practices rather than general CSR dimensions is considered significant.

Talent management and CSR are at a vital stage in their development. However, talent retention has received limited attention and this area necessitates further exploration. Nevertheless, studies regarding the impact of organizations’ sustainable practices were inconclusive. Vinerean et al. (2013) gave evidence that a company’s CSR activities comprised an increasingly important way to attract and retain good employees. Meanwhile, Leveson and Joiner (2014) study about the importance of organizational CSR in choosing a job, showed that in spite of considering CSR as an important factor, the majority preferred greater extrinsic benefits on CSR criterion in the job choice process. Furthermore, Ohlrich (2015) suggested that it is not necessarily because of the CSR program that will make the talent stays in the organization. Furthermore, higher corporate social performance (CSP) increases the organization attractiveness as a result of their organization’s positive reputation (Greening and Turban, 2000). Albinger and Freeman (2000) suggested that CSP’s relationship with organization attractiveness depended on a job seeker’s specific situation.

In line with the recent Gond et al. (2017) review indicated that mechanisms and boundary conditions that can explain individual reactions to CSR practices are underexplored. The current study argues that the relationship between CSR practices and talent retention as other similar relationships cannot be considered to be universal and that contextual and individual factors will provide some meaningful boundary conditions on its impact. Therefore, it explores the black boxes of how CSR practices affect talent retention. This is performed by incorporating mediating and moderating mechanisms underlying this relationship, such as how employees’ attitudes (motivation, trust) mediate the CSR-talent retention relationship and how the moderating effect of other-regarding value orientation (ORVO) on both the direct and the indirect CSR-talent retention relationships.

Previous researchers have also suggested including attitudes as a mediating variable (Farooq et al., 2014). Kundu and Gahlawat (2016a) found that employees’ perceptions of socially responsible HRM have a significant impact on their attitudes. The results obtained by Chitsaz-Isfahani and Boustani (2014) indicated that there is a significant relationship between talent management, employee retention, and organizational trust.

Although socially responsible HRM practices may evoke trust and motivation among talented employees, the effects may vary according to individual differences of the talented employees such as ORVO (Rawshdeh et al., 2018). The role of individual differences as a moderator of the relationship between CSP and talent job pursuit intentions was proposed by Greening and Turban (2000) as a competitive advantage in attracting a quality workforce. Furthermore, embracing organizational values is often required from employees in their work behavior even if they personally do not uphold them (Gruys et al., 2008; Bridoux and Stoelhorst, 2016). High-level other-regarding value employees make sense of their organization’s care about stakeholders behind profit maximization and correspondingly take action (Burke et al., 1992). In this study, the focus is on ORVO as a moderator of the socially responsible HRM practices-talent retention direct relationship, as well as on the mediation of both trust and motivation. Accordingly, it is assumed that perceived SRHRM practices–talent retention direct relationship, as well as the mediation of both trust and motivation, are stronger for individuals who have high other-regarding value as compared to those who have low other-regarding value.

In sum, perceived socially responsible HRM practices may have favorable effects on talent retention. On the basis of previous studies and theories, this relationship may be mediated by employees’ attitudes (trust, motivation). In addition, the strength of the direct relationship between SRHRM practices and talent retention, as well as the mediation of both trust and motivation, may depend on ORVO. This study provides four main contributions: First, as organizations are facing mounting pressure to be more efficient and effective in utilizing their resources, it is fundamental to identify the optimum strategy that invests in sustainability dimensions and maximizes its positive impact on employees in general and on talented ones in particular. Therefore, this study brings a new perspective to advance the knowledge in talent management. It is suggested that perceived socially responsible HRM practices can be used to enhance employees’ impression of intrinsic rewards, instead of relying solely on financial benefits. Furthermore, using CSR/sustainability to motivate employees is more challenging to imitate than offering higher financial rewards.

Second, the major contribution of this study would be exploring the black boxes of how CSR affects talent retention. This is carried out by incorporating important mediating and moderating mechanisms underlying this relationship. They include how employees’ attitudes (motivation, trust) mediate the link between perceived SRHRM and talent retention. Furthermore, how ORVO moderates both the direct perceived SRHRM-talent retention relationship also the indirect mediation effect of perceived SRHRM on talent retention through (a) motivation and (b) trust.

Third, this study will contribute to the current literature by covering that gap and responding to requests for studies on how CSR influences employees’ level. Furthermore, the current study expands the CSR literature through the HRM scope.

Fourth, this study contributes to the body of literature by providing further insight into the relationship between CSR and talent management in other cultural contexts, an Eastern context (Malaysia). This warrants further valuable contribution from a contextual perspective since Malaysia is a rapidly growing developing country and is exploring the CSR-talent management relationship.

## 2. Literature review

### 2.1. Talent management and retention

Talent management has been widely acknowledged to be one of the most essential factors for organizational competitive advantage (Rose and Kumar, 2006; Collings and Mellahi, 2009). Scholars have demonstrated that effective talent management is positively related to organizational success (Asrar-ul-Haq and Anwar, 2018) performance (Glaister et al., 2018), and also individual outcome variables (Bethke-Langenegger et al., 2011). Talent management focuses on attracting, recruiting, rewarding, and retaining talent (Lewis and Heckman, 2006).

Talent retention is conceived as an essential element that elicits the performance, development, success, and existence of the organizations (Wheelock, 2010). Frank et al. (2004) defined talent retention as the organization’s attempts in holding its desirable employees toward achieving its objectives. Moreover, talent turnover has a high financial cost which might exceed 200% of their yearly salary (Cascio and Boudreau, 2010; Mihelic and Plankar, 2010). Talent turnover can result in the loss of tacit knowledge, work disturbance, and might propel other valued employees to quit the organization (Allen et al., 2010).

During the past 5 years, Talent Corporation, an organization formed by the Malaysian government to handle talent development and management initiatives, has developed several transformation programs to encourage Malaysian experts abroad to return (Malaysia, 2012). Surprisingly, Malaysia is still facing the phenomenon of Brain Drain from 1995 to 2013 with more than a million skilled workers migrating from Malaysia, despite the Malaysian Government spending millions of dollars on diaspora activity abroad (Lim et al., 2014). To date, there are no reliable statistical data on SRHRM available for Malaysia to indicate the prevalence of this work issue.

#### 2.1.1. Factors for retention

Varied factors affect talent retention, located at organizational (macro) level and employee (micro). To start with, Gordon and St-Amour (2000) demonstrated ten factors that influence retaining talent. They are talent fit with corporate culture, development for their skills, joint responsibility and attachment, positions match their competencies, training, motivation meeting their need, instant financial bonuses, involved in the process of problem-solving in their corporate, and sense of togetherness. Furthermore, Carleton (2011) claimed that real satisfaction results from challenging and interesting jobs, not the reward level that maintains the talent. Furthermore, Ibidunni et al. (2016) discussed the effective impact of payment, promotion, recognition, supervisor/other-relationship, and physical working environment on retaining talent. On the other hand, Coombs (2009) indicated the relationship between organization identification, attitudes, and perceived behavioral control, with the intention to stay among IT professionals. Meanwhile, Osman-Gani and Paik (2016) identified career development programs, training opportunities, and skills development as the top factors associated with HR practices affecting the retention of IT talent. Regarding the importance of satisfaction to talent retention, Porter (2011) pointed out that conducting satisfaction studies is fundamental. Mihelic and Plankar (2010) mentioned that the lack of job challenge and satisfaction were the top factors mentioned for talent turnover.

#### 2.1.2. Techniques for talent retention

Techniques for talent retention have been adopted by HR officials and in this regard, different researchers shared their views and gave their experiences. In Lewis and Heckman (2006) view, talent management is made up of HR practices like selection, recruitment, development and training, employer branding, career management, and motivation. The strategies suggested by Lockwood and Ansari (1999) for retaining talent include mainly HR practices. In addition, developing and managing strong HR strategies for retaining talent are considered an integral part of HR responsibilities McDonnell (2011), following different HR generic strategies. In their article, Ali et al. (2000) considered the recruitment strategies like acquisitions, HRD, and employee referral as the major factors for talent retention. Furthermore, Zheng (2009) confirmed the significant effect of HR practices on corporate performance and talent retention, and suggested exploring talent management HR practices link in future research. Research on organizational behavior resulted in consistent findings about the positive impact of appreciation and attention for talented employees toward their performance motivation (Bethke-Langenegger et al., 2011).

In addition, the literature provided the key factors that affect talent retention such as job satisfaction and intrinsic motivation (Chiu et al., 2002), leadership development and succession planning (Ashridge, 2008), and employee engagement (Bhatnagar, 2007). However, despite the findings of Siwar and Hossain (2009) confirming that talent retention and development are the highest impacts of all talent investments on employee commitment and contribution, talent management research (including talent retention) from the individual perspective is scarce. Nevertheless, the positive impact on an individual variable outcome like talent motivation (Bethke-Langenegger et al., 2011) is limited.

Prior literature shows that talent retention plays an important role in organizational success and performance, and is a key driver to sustainable competitive advantage. Moreover, the literature claims that retaining talent is getting more difficult. In addition, it indicates both the financial and non-financial costs of talent turnover. Furthermore, the literature provided varied HR practices, strategies, job engagement techniques, and financial and non-financial rewards for retaining talent. However, talent management research (including talent retention) from the individual perspective is scarce. Although talent retention is an emerging issue, it still needs more investigations to show its comprehensive image.

### 2.2. Corporate social responsibility

Jamali and Mirshak (2007) define the social responsibility of business as the economic, legal, ethical, and discretionary expectations that society has of organizations at a given point in time. CSR practices impact the organizational performance, image, and reputation (Burke et al., 1992; Rupp et al., 2013; Newman et al., 2016) as well as internal and external stakeholders such as employees, customers, and owners (Li et al., 2013; Story and Neves, 2015). The Malaysian government has required all listed companies in bursa Malaysia to include their CSR information in their annual disclosure report since 2007. Furthermore, the Malaysian government has incorporated CSR as an integral part of achieving both Malaysia’s vision for 2020 and the strategic objectives of the National Integrity Plan (Hamid and Atan, 2011). Moreover, multiple awards have been established to encourage CSR as Prime Minister Awards, ACCA Malaysia Sustainability Reporting Award (ACCA MaSRA), Ansted Social responsibility International Award (ASRIA), StarBiz-ICR Malaysia Awards, and Social Reporting Awards.

The available CSR studies in Malaysia mostly concentrate on the aspects of disclosure and reporting through organizations’ websites and annual reports (Nasir et al., 2015). On the other hand, limited studies have covered stakeholder awareness and perception. They were focused on the Islamic context showing the consistency between CSR orientations and Islamic manners (e.g., Altruistic, ethical and helpful) (Ilyas, 2018). To date, there are no reliable statistical data on SRHRM available for Malaysia to indicate the prevalence of this work issue. This study contributes to the body of literature by filling that gap and providing further insight into the relationship between CSR and talent management in an Eastern context (Malaysia). Moreover, Malaysian firms are increasingly implementing CSR practices in order to attract and motivate talented employees. Nasir et al. (2015) had called for more studies in the Malaysian context on awareness and perception toward CSR. Hence, more research in emerging economies is to be carried out, where talent retention is particularly crucial (Vaiman et al., 2012). In addition, it provides further exploration into the CSR-talent management relationship in other cultural contexts (Kundu and Gahlawat, 2016a).

## 3. Hypotheses and conceptual framework

The following sections demonstrate the hypothesized relations between the variables in this study and the overall hypotheses development.

### 3.1. Perceived socially responsible HRM and talent retention

Socially responsible human resource management refers to a set of HRM practices followed by organizations to impact employees’ behaviors and attitudes in the process of assisting external CSR practices implementation (Shen and Benson, 2016). Kundu and Gahlawat (2015) redefined SRHRM as a set of HR activities designed to improve employees’ involvement in CSR while accounting for them both as an implementer and beneficiaries of CSR. Kundu and Gahlawat (2016a) have indicated that all the four components of SRHRM, such as legal compliance HRM, employee-oriented HRM, general facilitation HRM, and general CSR conduct, have effects on employees’ work-related attitudes, namely trust, motivation, and affective commitment. When CSR merges with HRM, it improves the existing knowledge foundation of both HRM and CSR (Bučiūnienė and Kazlauskaitė, 2012; Manroop et al., 2014; Newman et al., 2016; Cohen, 2017; Rawshdeh et al., 2018).

Multiple studies have linked employees’ perceptions of their organization’s ethical climate (processes and context) to positive employee work outcomes and turnover intentions through meeting their developmental and ideological job needs and involving the employees in the process of implanting CSR in their culture (Du et al., 2010; Kontoghiorghes, 2016; Heinrich, 2017).

de Jong (2011) explored that employee-oriented CSR practices promote employees’ identification and commitment that results in reducing absenteeism and turnover rate as well as enhancing creativity and productivity, in addition to the significant importance of both internal and external CSR dimensions regarding employee retention (Aminudin, 2013). Furthermore, the employees’ recognition of their organization’s CSR engagement and development rewarding programs for that can increase their pride and loyalty toward their organization, and this directly reinforced retention (Yim and Fock, 2013).

Thompson and Bunderson (2003) demonstrated that implementing CSR through human resource practices might be a significant element for employer–employee ideological psychological contracts and therefore, it improves employees’ commitment and contributions to their firm. Furthermore, Kundu and Gahlawat (2015) drawing on the literature, the following hypothesis can be proposed:

Hypothesis 1: There is a positive impact from perceived SRHRM on talent retention.

### 3.2. Mediating role of motivation on the relationship between SRHRM practices and talent retention

The literature shows that more than hundred attempts to define motivation have been made (Meyer et al., 2004). Latham and Pinder (2005) definition states that motivation represents a set of energetic forces that induce individual work-related behavior and determines its form, direction, intensity, and duration. Motivation is a practice through which behavior is manipulated, sustained, and energized for organizational objectives (Ryan and Deci, 2000; Deci and Ryan, 2008). Although empirical evidence has shown that SRHRM practices positively influence employees’ outcomes, knowledge on the social impact of SRHRM practices on employee’s wellbeing is limited (Zhang et al., 2022).

Empirical research showed the positive effect of employees’ perceptions of their organization’s socially responsible practices on their outcomes such as identification, loyalty, commitment, trust, and satisfaction (Brammer et al., 2007; Rupp et al., 2013; Rawshdeh et al., 2019). Melynyte and Ruzevicius (2008) a survey that was conducted in 25 countries supported the idea that organizations’ engagement in social responsibility enhanced the employee’s motivation and loyalty. Different studies showed that the issue of employees’ motivation trust and affective commitment serves to be one of the main benefits of organizations’ CSR engagement, with their results interpreted using social identity theory (SIT) (Kim and Scullion, 2013; Kundu and Gahlawat, 2016b). Research has shown that intrinsic motivation and Mastery-approach goals are strongly and negatively related to turnover intention (Kundu and Gahlawat, 2015). Taken together, these studies showed that motivation serves as an antecedent for retaining employees. Kontoghiorghes (2016) ascertained that high-performance organizational culture predicted talent attraction and retention through the mediation of employees’ satisfaction, motivation, and organizational commitment. The study relied on and was consistent with, P-O fit theories that indicate the effect of high (performer–performance organization) fit on high-performer attitudes (Rawshdeh et al., 2019).

Social exchange theory (SET) is an influential conceptual paradigm for understanding workplace behavior (Blau, 1964; Gergen, 1969). The SET suggests that when an organization’s treatment of its employee is fair and provides them with socio-emotional or economic resources (Gould-Williams, 2007), the employees in turn feel obliged to react to their organization’s positive treatment of them through a positive attitude that directly benefits the organization such as trust and motivations. This employee’s perception might be explained in two ways: first, when CSR practices impact the employees directly, then in turn, it would justify why they would choose to reciprocate positively to their organization. Second, even when CSR practices are not directly oriented toward employees’ benefits, employees may link these CSR practices with their values, beliefs, and morals; and as a result, employees may feel that they should reciprocate positively in response to the organization’s valued CSR practice (Cropanzano and Rupp, 2008). In HRM literature, SET illustrated how organizations’ HRM practices drive their employees to promote their organizational identification level and to reciprocate with positive attitudes and behaviors toward their organization’s benefits (Brammer et al., 2007). It provides an explanatory structure that explains the link between employees’ perceptions regarding their organizational SRHRM practices and their turnover intentions and job satisfaction (Kundu and Gahlawat, 2016a). SRHRM effect on employees’ work attitudes can be theoretically explained using the SET and SIT (Newman et al., 2016).

Social identity theory emphasizes the influence of membership in different social organizations, as the organization for which individual works, on the individual’s self-concept (Tajfel, 1978; Dutton et al., 1994). SIT explicitly prescribes constructs such as respect, prestige, distinctiveness, and organizational identification as focal variables (Ashforth et al., 2008). Based on SIT, employees are supposed to view an organization’s identity as more attractive as it matches their own self-aspect (Hogg and Turner, 1985). Individuals incline toward identifying organizations when they sense a notable overlap between organizational and individual attributes, when they perceive the organization’s high prestige and attractive image and when organizational identity can enhance the members’ self-esteem (Ashforth and Mael, 1989; Pratt, 1998). SIT is a highly influential theory for understanding and predicting CSR at the micro-level by Rupp et al. (2013), Jones et al. (2014), it explains why organization’s CSR initiatives influence its employees (Sen and Bhattacharya, 2001).

Previous research relies on SIT to explain the direct relation between CSR and commitment and developed models of the impact of perceived CSR on organizational identification (Glavas and Godwin, 2013). Kim et al. (2010) proposed that the effect of CSR on AOC by the mediation mechanism of employees’ organizational identification stays in conformity with the social identity framework. Peterson (2004) expected CSP to contribute positively to the attraction, retention, and motivation of employees because they are likely to identify strongly with positive organizational values according to SIT. Drawing on these research findings, the next hypothesis can be proposed:

Hypothesis 2: Motivation will mediate the impact of perceived SRHRM on talent retention. Specifically, (H2a) perceived SRHRM is related positively to motivation, and (H2b) motivation is related positively to talent retention.

### 3.3. Mediating role of trust on the relationship between socially responsible HRM and talent retention

Trust has also been defined as the belief of the employee in the fact that the organization will maintain the commitments, negotiate honestly and would not take excessive advantage (Bies and Tripp, 1996). The current study defines trust as “one’s willingness to be vulnerable to the actions of another party based on the expectation that the other party will perform a particular action important to the trustor, irrespective of the ability to monitor or control that other party” (Mayer et al., 1995). An organization’s ethics and values are regarded as a source for stakeholders’ trust (Mayer et al., 1995; Turker, 2009). Employees consider CSR activity as an important signal about the organization’s ethics and values (Rupp et al., 2013). Hence, Cornelissen et al. (2007) suggested that stakeholders, including employees, regarded organizations engaging in CSR practices act as “trustees.”

Studies have argued that highly trusted organizations drive positive behaviors and attitudes starting from increasing job pursuit intention for prospective employees, to a higher level of employees’ commitment, satisfaction, organizational identification and even reducing turnover intention (Greening and Turban, 2000; Farooq et al., 2014; Kundu and Gahlawat, 2016a). Trust, culture, and satisfaction impact an employee’s commitment and his or her intent to stay with the organization indicating the significant relationship between talent management, employee retention, and organizational trust (Chitsaz-Isfahani and Boustani, 2014). Employee trust has repeatedly been negatively related to turnover intentions (Hemdi and Nasurdin, 2006). Hansen et al. (2011) highlighted the role of organizational trust in mediating the relationships between CSR and turnover intentions and OCB. In addition, Farooq et al. (2014) showed that the effect of CSR on affective organizational commitment is fully mediated by both organizational trust and organizational identification. Thus, we propose this hypothesis:

Hypothesis 3: Trust will mediate the impact of perceived SRHRM on talent retention. Specifically, (H3a) perceived SRHRM is related positively to trust, and (H3b) trust is related positively to talent retention.

### 3.4. Moderating role of as other-regarding value orientation on the socially responsible HRM–talent retention relationship

Thus far, the direct and mediation routes from SRHRM have been proposed, respectively. However, despite these propositions and their theoretical rationalization, we expect their effect to not be absolute for all talented employees. Hence, we predict that individual differences, which are a primary source for self-concept, and self-worth and accordingly, generate the employee’s identification, may act as boundary conditions on the direct and the mediation links hypothesized previously in this study.

Other-regarding value orientation refers to individual differences that are the extent or degree to which an individual considers being compassionate and helpful to others and caring about others’ welfare as important (De Cremer and Van Lange, 2001). These individual differences impact their behavior when associating with others (Bridoux and Stoelhorst, 2016). Other-regarding individuals’ behavior of caring for the fairness and outcomes of others and as well as for their own is underpinned by reciprocity norm, as when individuals perceive that others are behaving (un)fairly, they will react in increasing (decrease) the outcome for that other party (De Cremer and Van Lange, 2001). Embracing organizational values is often required from employees in their work behavior even if they do not promote those values (Gruys et al., 2008; Nishii et al., 2008). Under these circumstances, and according to the supplies–values fit theory, negative employee consequences are likely to result from the supplies–values misfit such as job dissatisfaction, employee compromised work performance, and the intention to quit or even the actual turnover (Edwards and Lambert, 2007). Therefore, gaining employee fit with policies and practices that demonstrate organizational values is important for the organization and will affect both employees and the organization.

Talented employees may value SRHRM practices because they provide a forecast for an organization’s future treatment of its employees, which offer a feeling of comfort expectation regarding their material outcomes (Farooq et al., 2014; Jones et al., 2014). Furthermore, it fulfills their need for being compassionate and helpful to other stakeholders outside and inside the organization (Rupp et al., 2013). CSR impacts the organization’s reputation and image leading to a favorable social identity (Rupp et al., 2013; Jones et al., 2014). Beyond the material and relational self-serving benefits that SRHRM practices provide, talented employees may value SRHRM practices because, from a moral standpoint, fair treatment of others is the right thing (Rupp et al., 2013).

This moderator connects directly to the SIT foundation in this study as well as our target to understand the channels in which SRHRM influences talent retention through more inwardly and outwardly focused judgments that serve to catalyze more inwardly and outwardly behaviors, respectively.

This study suggests that ORVO will strengthen the impact of perceived SRHRM on talent retention. Values work as a schema that influences the saliency of climate information (Martin et al., 2000). Talents with high levels in ORVO are likely to be attentive to the SRHRM practices of their organization. Working for an organization that has SRHRM practices provides employees the avenues to act consistently with personally favorable modes of conduct. This study predicts a moderated mediation effect, expecting the strength of the mediated impact to differ at varying degrees of the proposed moderator. Particularly, we expect the indirect impact of perceived SRHRM on talent retention (through the mediation of trust, and motivation) to be stronger for employees with high ORVO. This assumption is compatible with former scholars’ debates in the CSR literature, such as (Riordan et al., 1997) proposition that different people selectively process different informational signals relevant to their social responsibility, according to the relevance of that information to them. Evans et al. (2011), Bridoux and Stoelhorst (2016), and Valentine and Godkin (2017) found that perceived corporate citizenship (PCC) significant positive impact on OCB was stronger for high ORVO employees, also that a high degree of ORVO strengthened the positive link between PCC and organizational identification.

Based on the previously mentioned arguments, this study suggests that employees process information more selectively, aligned with their social orientation, and thereby, they are fully appreciative of the particular forms of SRHRM as they influence the retention processes. This intensifies the hypothesized positive effect of SRHRM practices on talent retention. Hence, we predict that ORVO will moderate the impact of perceived SRHRM on talent retention. Moreover, as motivation and trust mediate the impact of perceived SRHRM on talent retention, we predict that ORVO also moderates the indirect mediation impact of perceived SRHRM on talent retention through (a) motivation and (b) trust. Accordingly, we propose Hypothesis 4.

Hypothesis 4: Other-regarding value orientation moderate the relationship between perceived SRHRM and talent retention.

Hypothesis 5: Other-regarding value orientation moderate the indirect mediation impact of perceived SRHRM on talent retention via (a) motivation and (b) trust.

### 3.5. The conceptual model

**FIGURE 1:**
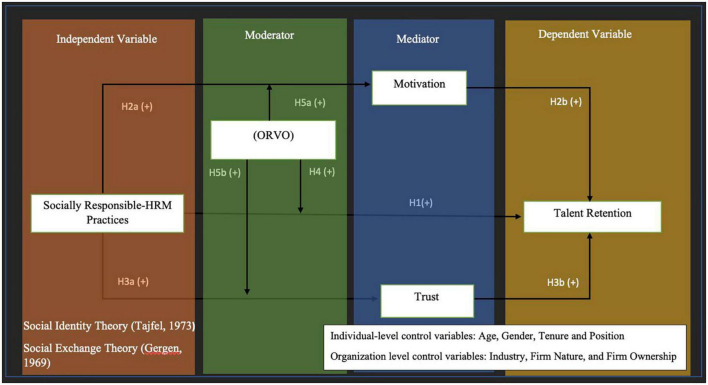
The conceptual model.

Figure 1 shows the hypothesized relationships among study variables using a mediation-moderation framework. As shown in the figure, SRHRM is both directly and indirectly related to talent retention. Trust and motivation act as mediating mechanism that explains the relationship between SRHRM and talent retention. Specifically, ORVO is predicted to moderate the impact of perceived SRHRM practices on talent retention as well as to moderate the mediation of both motivations.

## 4. Sample and data collection

This study examines the effects of perceived SRHRM practices, employees’ attitudes (motivation, trust), and ORVO on talent retention. This study examines a predictive model which is developed based on existing theories and is undertaken cross-sectionally through talented employees from Malaysia. The participants’ responses were extracted by distributing self-reported questionnaires. The University of Kebangsaan Malaysia provided a letter of collaboration for studies where students’ opinion is needed. The letter is provided in the appendix. The consent was included on the first page of the survey by telling the participant that it is voluntary to take part and that they can stop at any time. MBA students and alumni with at least 1 year of working experience as a proxy to measure talents were used as respondents. According to Ceja Barba and Tàpies (2009), MBA students are highly talented potential employees who can be a source of competitive advantage for organizations. The selection to represent talents is also consistent with several earlier studies by respectful scholars and their studies published in respectful management journals. Purposive sampling was employed in this study because the study obtains information relevant to, and available only to, a certain group, that is, MBA students and alumni with at least 1 year of working experience. However, it is hard to reach all talented employees who are working in Malaysian organizations. Therefore, the non-probability sampling technique is used since an accurate sampling frame cannot be provided. Furthermore, the focus of this study is on theory generalization and not population generalization (Highhouse, 2009).

The main study variables, which are socially responsible HRM practices, motivation, trust, ORVO, and talent retention, were measured by adopting measurement scales that have been used and validated previously in established journals. For those constructs that fit the study purpose, the measurement items were adopted from established measurements used in past studies, and the measurement scale used was the five-point Likert-type scale. However, the measurement items were adopted with slight modifications that were necessarily based on expert interviews conducted during the validity to further capture the study background. Table 1 shows the number of measurement items used for every variable in this study model.

**TABLE 1:** Measurement items for the study constructs.

Construct	Number of items	Source
Perceived socially responsible HRM	6	Shen and Benson’s (2016)
Trust	7	Robinson and Rousseau (1994)
Talent retention	4	Kyndt et al. (2009)
Other-regarding value orientation	4	Roman et al. (1999)
Motivation	6	Kuvaas and Dysvik (2009)

A total of 700 survey questionnaires were distributed manually. A total of 460 surveys were returned within 1 month. Out of the questionnaires returned, 15 questionnaires did not meet the proposed criteria and 25 questionnaires had not been fully completed. Accordingly, 420 questionnaires were usable for further analyses as they were fully answered, and the participants fit the criteria (MBA) students and alumni in the Klang Valley area in Malaysia with a minimum of 1 year of working experience.

## 5. Results and discussion

Partial least square (PLS) path modeling was used to test the theoretical model. For determining the minimum acceptable sample size for PLS-structural equation mode (SEM), two ways were used: first, we have the G Power test, which is a statistical measure usually used in the social, behavioral, and biomedical sciences conducted to identify the appropriate sample size and to avoid the presentation of non-significant results during the analysis stage (Faul et al., 2007). The result of the G Power test to determine the correct sample size for this study is 119 respondents. Second, (Hair et al., 2014) the sample size was recommended by Hair et al. (2014) when using PLS-SEM.

### 5.1. Measurement model

**FIGURE 2:**
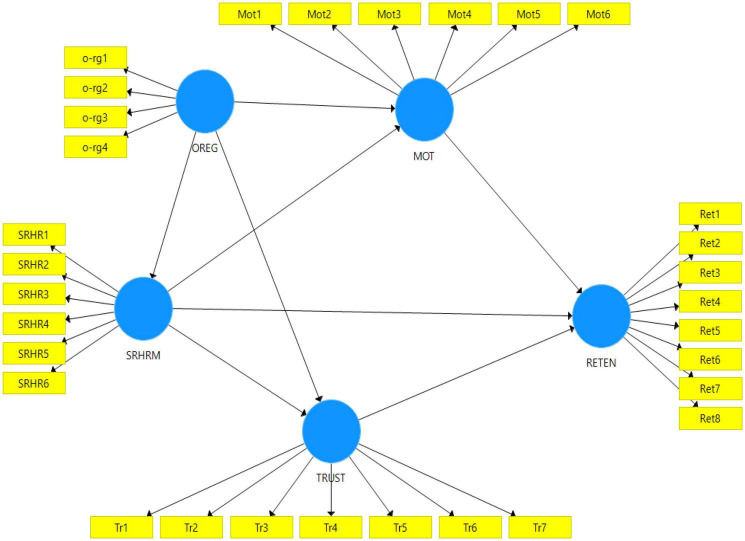
Measurement model.

To assess the reflective measurement model in PLS, the independent variables perceived SRHRM consisting of six indicators (SRHRM1–SRHRM6), mediators trust [consisting of seven indicators (Tr1–Tr7)], motivation consisting of six indicators (Mot1–Mot6), the moderator other regarding values [consisting of four indicators (o-rg1–o-rg4)], and the dependent variable talent retention [consisting of eight indicators (Ret1–Ret8)] connected in the model as displayed in Figure 2. Then, the PLS algorithm was calculated.

**FIGURE 3:**
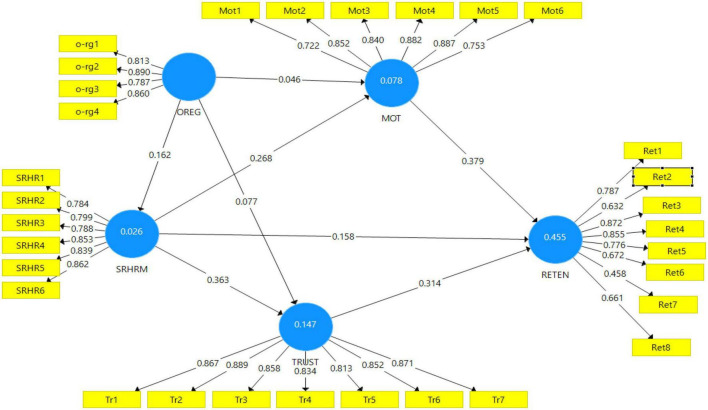
Outer loadings.

An evaluation of the measurement model starts with convergent validity, which is achieved by four criteria, factor loading, Cronbach’s alpha (CA), then, composite reliability (CR), and average variance extracted (AVE). Next, the discriminant validity is achieved by cross-loading, the Heterotrait–Monotrait ratio of correlations (HTMT), and the Fornell–Larcker criterion. The reason for assessing indicator reliability is to evaluate the extent to which an indicator is consistent with what it aims to measure (Urbach and Ahlemann, 2010). The outer loadings assess the correlation of the latent construct and its respective items, where its values must be greater than 0.70 to be capable of explaining at least 50% of indicator variance (Hair et al., 2021). Outer loading below 0.40 means that the indicators must be deleted, whereas an indicator with outer loading between 0.40 and 0.70 is deleted if the deletion will increase the average variance composite or extracted reliability, higher than the suggested value. In the current study, the outer loadings for all indicators were higher than 0.70, except for Ret7 = 0.456, Ret8 = 0.661, Ret6 = 0.672, and Ret2 = 0.632 as shown in Figure 3.

**FIGURE 4:**
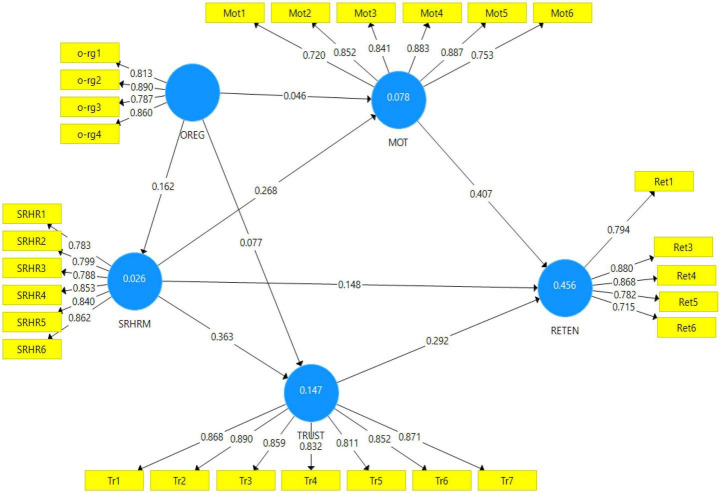
The measurement model after modification.

The items were deleted one at a time, starting with the lowest loading (Ret7 = 0.456), followed by the PLS algorithm was calculated again to ensure that the constructs achieved the required threshold value of internal consistency, (Hair et al., 2021). Items were deleted one by one to check whether the loading of the other items could improve the AVE result. Accordingly, items labeled as Ret7, Ret8, and Ret2 were deleted one at a time. After the above-mentioned items were deleted from the model, the internal consistency results for every construct were in the suggested acceptable range. Figure 4 shows the measurement model after modification.

Average variance extracted is identified as the level to which the latent construct can explain the variance of its indicators (Hair et al., 2014) and should be equal to, or more than, 0.50 (Hair et al., 2021). CA measures the intercorrelation between the indicators. The most widely used cut-off criterion for CA is 0.70 (Nunnally, 1994). The CR score measures individual reliability and provides a good estimate of the variance shared by respective indicators in the PLS-SEM model (Hair et al., 2014). The satisfactory value indicates that the internal consistency or adequate convergence CR is 0.7 or above (Ramayah et al., 2016). Table 2 shows the factor loading, CA, CR, and AVE. It shows that all the values were above the mentioned cut-off points. Accordingly, convergent validity and construct reliability are achieved.

**TABLE 2:** Convergent validity.

Construct	Item	Outer loading	Cronbach’s alpha	Composite reliability	AVE
SRHRM	SRHRM1	0.783	0.903	0.925	0.674
	SRHRM2	0.799			
	SRHRM3	0.788			
	SRHRM4	0.853			
	SRHRM5	0.840			
	SRHRM6	0.862			
Motivation	Mot1	0.720	0.905	0.927	0.681
	Mot2	0.850			
	Mot3	0.841			
	Mot4	0.883			
	Mot5	0.887			
	Mot6	0.753			
Trust	Tr1	0.868	0.939	0.950	0.731
	Tr2	0.890			
	Tr3	0.859			
	Tr4	0.832			
	Tr5	0.811			
	Tr6	0.852			
	Tr7	0.871			
ORVO	o-rg1	0.813	0.863	0.900	0.692
	o-rg2	0.890			
	o-rg3	0.787			
	o-rg4	0.860			
Talent retention	Ret1	0.794	0.867	0.905	0.656
	Ret 3	0.880			
	Ret4	0.868			
	Ret5	0.782			
	Ret6	0.715			

Discriminant validity indicates the relationship between the latent construct and other constructs in the model. In PLS-SEM, discriminant validity might be assessed by different tests, for example by testing the cross-loadings of the indicators, Fornell and Larcker and the HTMT. Outer loading must be above 0.708 (Hair et al., 2014), and the indicator’s outer loading should be greater than the cross-loadings of all the other constructs in the same line. This is because the constructs can face a discriminant problem in case the indicator’s outer loading is smaller than the value of the cross-loadings of the other latent constructs. The difference between the loadings among the latent variables must be equal to or higher than 0.1 (Chin, 1998). It is also crucial to note that each indicator should load low on other constructs but high on its own constructs (Ramayah et al., 2016).

Fornell and Larcker (1981) state that a latent variable should be able to describe the variance of its items better than it describes the variance in the other latent variables. Therefore, the latent variable’s AVE should be greater than the squared correlation between the latent variables and other variables (Chin, 1998). The result shown in Table 3 indicates that the criteria have been met.

**TABLE 3:** Fornell–Larcker criterion result.

	MOT	OREG	RETEN	SRHRM	TRUST
MOT	0.825				
OREG	0.097	0.832			
RETEN	0.592	0.114	0.810		
SRHRM	0.274	0.158	0.371	0.821	
TRUST	0.494	0.140	0.549	0.374	0.855

MOT, motivation; OREG, other-regarding value orientation; RETEN, retention; SRHRM, strategy human resources management.

The HTMT is the ratio of correlations within the constructs to correlations among the constructs. A significance level of *p* = 0.05 (at a 95% confidence interval) is suggested by Henseler et al. (2016). An HTMT value close to 1 shows a lack of discriminant validity. The result as shown in Table 4 indicates that the discriminant validity is achieved based on HTMT criteria.

**TABLE 4:** Heterotrait–Monotrait ratio of correlations (HTMT) results.

	MOT	OREG	RETEN	SRHRM	TRUST
MOT					
OREG	0.097				
RETEN	0.656	0.116			
SRHRM	0.305	0.173	0.421		
TRUST	0.539	0.141	0.603	0.408	

MOT, motivation; OREG, other-regarding value orientation; RETEN, retention; SRHRM, strategy human resources management.

### 5.2. Structural model

In the first stage of assessing the structural model, it is important to ascertain the variance inflation factor (VIF) values of the independent variables to determine whether there is lateral collinearity in the model. All the inner VIF values of the independent variables should be less than 5.0 or less than 3.3 (Hair et al., 2011). As shown in Table 5, all the inner VIF values meet both cut points indicating that there is no collinearity concern (Hair et al., 2017).

**TABLE 5:** Result of inner VIF values.

	Motivation	O reg	Talent retention	SRHRM	Trust
Motivation			1.340		
O reg	1.027			1.00	1.027
**Talent retention**					
SRHRM	1.027		1.179		1.027
Trust			1.442		

Path coefficients (p) represent the hypothesized relationship that links the construct. Its values are from −1 to +1; when the value is closer to +1 that means a positive strong relation, whereas it will be a strong negative relation when it is close to −1. To test the significant standard error, the (*t*-value) must be achieved using bootstrapping in SmartPLS (Hair et al., 2017). The critical values of significant level for *p* < 0.01 at *t*-values > 2.58 (two-tailed) at *t*-values > 2.33 (one-tailed), and for *p* < 0.05 at *t*-values > 1.96 (two-tailed) at *t*-values > 1.65 (one-tailed) (Hair et al., 2017).

Overall, this study shows that five direct hypotheses are developed between the constructs H1, H2a, H2b, H3a, and H3b. Based on the results in Table 6, the five relationships are found to have *t*-value > 2.33; therefore, it is significant at a 0.01 level of significance. Specifically, the predictors SRHRM (β = 0.139, *p* < 0.01), Motivation (β = 0.378, *p* < 0.01), and Trust (β = 0.298, *p* < 0.01) are positively related to talent retention, which explains 47.8% of the variances in talent retention. SRHRM is positively related to Motivation (β = 0.294, *p* < 0.01) and Trust (β = 0.360, *p* < 0.01). Thus, H1, H2a, H2b, H3a, and H3b are supported. The *R*^2^-value of 0.478 is above the 0.26 value as suggested by Cohen (1983), which indicates a substantial model.

**TABLE 6:** Results of structural model analysis (hypotheses testing).

Hypothesis	STD Beta	STD error	*t*-value	*P*-value	Confidence interval		*f* ^2^	VIF	Decision
					LL	UL			
H1	0.139	0.04	3.461	*p* < 0.001	0.073	0.199	0.039	1.286	Supported
H2	0.094	0.025	3.804	*p* < 0.001	0.056	0.137			Supported
H2a	0.249	0.055	4.552	*p* < 0.001	0.154	0.337	0.065	1.047	Supported
H2b	0.378	0.049	7.777	*p* < 0.001	0.298	0.454	0.218	1.39	Supported
H3	0.107	0.021	5.03	*p* < 0.001	0.073	0.143			Supported
H3a	0.36	0.05	7.171	*p* < 0.001	0.261	0.429	0.155	1.02	Supported
H3b	0.298	0.048	6.261	*p* < 0.001	0.219	0.38	0.129	1.469	Supported
H5a	0.106	0.062	1.705	0.044	−0.253	0.136	0.013	1.037	Not supported
H5b	0.174	0.169	1.025	0.153	0.136	0.276	0.041	1.001	Not supported
H4	0.031	0.038	0.809	0.209	−0.035	0.096	0.002	1.057	Not supported

**p < 0.01, *t*-value > 2.33 (supported at one-tailed).

*p < 0.05, *t*-value > 1.645 (supported at one-tailed). ns, not supported.

Next, the effect size *f*^2^ is assessed in reporting and interpreting studies as both the substantive significance (effect size) and statistical significance (*p*-value) are essential results to be reported. The *f*^2^-values of 0.02, 0.15, and 0.35 indicate small, medium, and large effects, respectively (Cohen, 1983). In Table 7, the results show that SRHRM (0.039), motivation (0.218), and trust (0.129) have small, medium, and medium effects, respectively, in producing the *R*^2^ for talent retention. Furthermore, the results show that SRHRM has a small effect in producing the *R*^2^ for motivation (0.056) and a medium effect in producing the *R*^2^ for trust (0.155).

**TABLE 7:** Results of the structural model analysis (direct relation).

Hypothesis	STD Beta	Std error	*t*-value	*P*-value	Confidence interval		*f* ^2^	VIF	Decision
					LL	UL			
H1	0.139	0.04	3.461	*p* < 0.001	0.073	0.199	0.039	1.286	Supported
H2a	0.249	0.055	4.552	*p* < 0.001	0.154	0.337	0.065	1.047	Supported
H2b	0.378	0.049	7.777	*p* < 0.001	0.298	0.454	0.218	1.39	Supported
H3a	0.36	0.05	7.171	*p* < 0.001	0.261	0.429	0.155	1.02	Supported
H3b	0.298	0.048	6.261	*p* < 0.01	0.219	0.38	0.129	1.469	Supported

**p < 0.01, *t*-value > 2.33 (supported at one-tailed).

*p < 0.05, *t*-value > 1.645 (supported at one-tailed). ns, not supported.

In addition, the predictive relevance of the model is tested using the blindfolding procedure. When the *Q*^2^-value is above zero, the model has predictive relevance for the endogenous variable (Hair et al., 2017). All the *Q*^2^-values as shown in Table 8 for motivation (0.056), trust (0.103), and talent retention (0.288) are larger than 0, implying that the model has sufficient predictive relevance.

**TABLE 8:** Results of the structural model analysis (mediation).

Hypothesis	STD beta	STD error	*t*-value	*P*-value	Confidence interval		*f* ^2^	VIF	Decision
					LL	UL			
H2	0.094	0.025	3.804	*p* < 0.001	0.056	0.137			Supported
H2a	0.249	0.055	4.552	*p* < 0.001	0.154	0.337	0.065	1.047	Supported
H2b	0.378	0.049	7.777	*p* < 0.001	0.298	0.454	0.218	1.39	Supported
H3	0.107	0.021	5.03	*p* < 0.001	0.073	0.143			Supported
H3a	0.36	0.05	7.171	*p* < 0.001	0.261	0.429	0.155	1.02	Supported
H3b	0.298	0.048	6.261	*p* < 0.001	0.219	0.38	0.129	1.469	Supported

**p < 0.01, *t*-value > 2.33 (supported at one-tailed).

*p < 0.05, *t*-value > 1.645 (supported at one-tailed). ns, not supported.

The analysis result has shown that the two indirect effects of mediation in H2 and H3 β = 0.094, β = 0.107 are significant with *t*-values of 3.804 and 5.030. The indirect effect of 95% Boot CI Bias Corrected: (LL = 0.056, UL = 0.137) and (LL = 0.073, UL = 0.143), do not straddle a 0 in between indicating that there is mediation (Preacher and Hayes, 2004). Accordingly, we can conclude that the mediation effects are statistically significant. Adapting Hair et al. (2017) mediation analysis procedure, the indirect effects h2a, h2b, h3a, and h3b, and the direct effects H2 and H3 are all significant and positive, which imply that the mediation is complementary (partial mediation).

Three hypotheses were developed to test the moderating effect of other regarding value. The first hypothesis of the moderating effect of other regarding value predicted that the other regarding value will moderate the effect of perceived SRHRM on talent retention (H4). H4: ORVO will moderate the direct relationship between SRHRM practices and talent retention, such that the relationship will be stronger when the levels of ORVO are high. Based on the analysis result in Table 9, the finding indicates that other regarding value does not moderate the perceived SRHRM on talent retention (β = 0.031, *t*-value = 0.809, *p* = 0.209) not statistically significant. *f*^2^ = 0.002 is below the cut-off point of 0.02 for a small effect (Cohen, 1983). The indirect effect of 95% Boot CI Bias Corrected: (LL = −0.035, UL = 0.096) straddles a 0 in between. Accordingly, H4 is rejected.

**TABLE 9:** Results of the structural model analysis (moderation).

Hypothesis	STD beta	Std error	*t*-value	*P*-value	Confidence interval		*f* ^2^	VIF	Decision
					LL	UL			
H5a	0.106	0.062	1.705	0.044	−0.253	0.136	0.013	1.037	Not supported
H5b	0.174	0.169	1.025	0.153	0.136	0.276	0.041	1.001	Not supported
H4	0.031	0.038	0.809	0.209	−0.035	0.096	0.002	1.057	Not supported

**p < 0.01, *t*-value > 2.33 (supported at one-tailed).

*p < 0.05, *t*-value > 1.645 (supported at one-tailed). ns, not supported.

The second hypothesis of the moderating effect of other regarding value predicted that the other regarding value will moderate the indirect mediation effect of SRHRM practices on talent retention *via* (H5A) motivation and (H5B) trust. H5: ORVO will moderate the indirect mediation effect of SRHRM practices on talent retention through (A) motivation and (B) trust, such that the indirect impact will be stronger when the levels of ORVO are high. Based on the analysis result in Table 9, the finding indicates that other regarding value does not moderate the indirect mediation effect of SRHRM practices on talent retention neither through (H5A) motivation (β = 0.106, *t*-value = 1.705, *p*-value = 0.044, *f*^2^ = 0.013) The indirect effect 95 percent Boot CI Bias Corrected: (LL = −0.253, UL = 0.136) straddles a 0 in between nor (H5B) trust (β = 0.174, *t*-value = 1.025, *p* = 0.153, *f*^2^ = 0.041). Accordingly, H5a and H5b are rejected.

## 6. Theoretical implications

Talent management and CSR are at a vital level in their development. Yet, the scarcity of rigorous research considering the impact of CSR on talent retention and in the previous research is surprising. Therefore, this study covered a gap in the literature by investigating the impact of perceived SRHRM on talent retention. Accordingly, the current study contributed to the existing knowledge by giving further insights regarding the importance of CSR to talent management. Underpinning the effectiveness of the implementation of CSR programs is the adoption of SRHRM practices. This study explored how SRHRM practices benefit the organization by their favorable impact on talent retention. It enriches CSR knowledge by exploring the impact of perceived SRHRM on talent retention and their attitudes (trust and motivation). As a result, this study perceived that SRHRM practices are positively and significantly related to talent retention, and this relationship is mediated by trust and motivation. On the contrary, prior research found that CSR influenced employee trust and motivation. This study enriches those studies finding by supplying additional empirical proof that perceived SRHRM would also influence trust and motivation which, in turn, increases talent retention. Most significantly, in exploring the impact of perceived SRHRM on talent retention, this study has lightened the mechanisms through which perceived SRHRM practices promote talents to retain in their organization. Particularly, it encourages us to explore the impact of perceived SRHRM on retaining talents and to understand it under the theoretical umbrella of the SET and SIT. The results of this study that perceived SRHRM practices improve talent’s trust, motivation, and retention in their organization, explain why talented employees respond to perceived SRHRM practices through the social exchange theory. On the other hand, employees’ perception regarding SRHRM practices explains talent retention under the SIT. Adopting SRHRM practices shows that an organization can conform to social CSR norms and implement CSR initiatives successfully (Orlitzky et al., 2003; Orlitzky and Swanson, 2006). Accordingly, it is likely to increase employees’ identification, which is also suggested by the SIT to be related positively to employees’ work attitudes (e.g., Trust). Finally, the study finds that employee attitudes play an important mediating role; it helps explain the relationship between perceived SRHRM and talent retention. Specifically, trust and motivation are found to mediate the relationship between perceived SRHRM and talent retention. The study, however, fails to establish the moderating role of ORVO on the link between perceived SRHRM and talent retention.

## 7. Practical implementations

Considering the increasing importance of CSR and talent management as the source of competitive advantage for organizations and an essential priority for their leaders worldwide, in addition to the HRM literature on the influence of HRM practices on employee attitudes and behaviors, the results of this study provide a number of significant practical implications for companies in Malaysia as well as the global context striving to successfully retain their talents and implement CSR initiatives. First, the study findings suggest that Malaysian firms could benefit from CSR practices to enhance their talent retention. Specifically, the positive effects of talented employees perceptions regarding SRHRM practices on their attitudes and the intention to stay in their organization indicate the ample benefits that can be achieved through the investment in socially responsible HR practices. Second, to undertake this, it an imperative for organizations to (a) consider CSR values in the recruitment and selection of prospective employees, provide opportunities for CSR training, and consider social performance in appraisals, promotion, and rewards systems, (b) allow employees take an active part in their organization’s CSR communications and associated activities to improve their awareness of CSR, and (c) facilitate and provide the opportunity for employees to participate in CSR initiatives (for example, in projects concerning community development, poverty alleviation, and sustainable behavior). These practices can communicate organizational CSR values and change employees’ perceptions about their organization which improves talent retention in their current organization. Thus, it provides a concrete, actionable recommendation for how to formulate and implement adequate SRHRM policies and practices as they are not only significant for the implementation of CSR programs successfully, but also for retaining talents and improving their motivation and organizational trust. Finally, there are limits to using SRHRM as a talent retention criterion. As shown in this study, both high and low ORVO employees have higher intentions to retain in their organization when they perceive that SRHRM practices have been initiated by their organization. Therefore, other-regarding values may not be a good criterion to be considered by HR managers in formulating SRHRM practices if the purpose is to retain talented employees who are affected by SRHRM practices.

## 8. Conclusion

The findings of this study demonstrate that perceived SRHRM predicts talent retention directly and indirectly through trust and motivation. This proposes that talented employees who experiment with SRHRM practices are more likely to stay at their current organization. A plausible reason for such a relationship, as found in this study, is when the organization treats the employees fairly and when it provides them with socio-emotional or economic resources (Gould-Williams, 2007). The employees, in turn, feel obliged to react to their organization’s positive treatment of them through a positive attitude that directly benefits the organization such as trust and motivations that directly impact the staying intention in the organization. In addition, the adoption of SRHRM practices by an organization shows that this organization can conform to social CSR norms and implement CSR initiatives successfully. Accordingly, it is likely to increase employees’ identification, which is also suggested by the SIT to be related to employees’ work attitudes and behaviors positively (Turker, 2009) hence it increases, and with this, they feel highly motivated to perform their jobs and trust in their organization and retain in it. Based on the results of the study, managerial and theoretical implications were derived, theoretically by giving more insights regarding the importance of CSR to talent management. Underpinning the effectiveness of the implementation of CSR programs is the adoption of SRHRM practices. Practically, the study findings suggest providing concrete, actionable recommendations on how to formulate and implement adequate SRHRM practices and policies as they are not only significant for the implementation of successful CSR programs, but also to retain talented employees and improve their motivation and organizational trust. The limitations of this study include, employing the cross-sectional design, the fact that the survey questionnaire was self-reported, and that we had used a purposive sample. We need valuable directions for scholars in future studies such as replicating this study in various work settings in addition to extending this study by including other different mediator and moderator variables in testing the impact of SRHRM on talent retention. Consequently, it would also be interesting for future studies to examine how these different discrete attitudes mediate the impact of the perceived SRHRM on talent retention to substantiate the findings. By employing a relative importance analysis, future studies may also identify which of these attitudes (i.e., identification, commitment, and satisfaction) contributes the most to explaining the variance in talent retention. The findings from this study will help to develop a further understanding of the theoretical level and managerial level of issues linked with CSR and talent management.

## Data availability statement

The raw data supporting the conclusions of this article will be made available by the authors, without undue reservation.

## Ethics statement

Ethical review and approval was not required for the study on human participants in accordance with the local legislation and institutional requirements. The patients/participants provided their written informed consent to participate in this study.

## Author contributions

ZR, SS, and ZM: methodology. MR and ZM: software. ZR, ZM, MR, and SS: validation. ZR and ZM: formal analysis and writing the original draft preparation. MR and SS: investigation and writing—review and editing. ZR: resources and supervision. ZR and SS: visualization. ZM: project administration. All authors have read and agreed to the published version of the manuscript.
